# Comparison of distinct gut bacterial communities in different stage of prediapause and nondiapause larvae in *Loxostege sticticalis*

**DOI:** 10.3389/fmicb.2024.1469140

**Published:** 2024-10-14

**Authors:** Jianyu Wang, Hainan Chong, Dong Li, Shaowei Cui, Yanni Song, Jinyu He, Tingbei Bo, Dandan Zhang, Haijun Xiao

**Affiliations:** School of Grassland Science, Beijing Forestry University, Beijing, China

**Keywords:** gut bacteria, intestinal microbial communities diversity, prediapause, functional prediction, seasonal adaptation, *Loxostege sticticalis*

## Abstract

**Introduction:**

Symbiotic microorganisms in insects regulate multiple physiological functions, widely participating in nutrient metabolism, immune regulation, and crucial regulatory roles in development. However, little is known about how microbial factors might respond to the preparation of insect diapause.

**Methods:**

The gut bacterial communities of *Loxostege sticticalis* larvae induced at different photoperiod of long (LD16:8, nondiapause) and short (LD12:12, prediapause) daylength were compared, by 16S rRNA Illumina MiSeq.

**Results:**

A total number of 42 phylum, 78 classes, 191 orders, 286 families, 495 genera, and 424 species were identified in the intestinal bacterial community of *L. sticticalis* larvae. Alpha diversity and beta diversity analyses revealed significant differences between nondiapause and prediapause larvae. In non-diapause larvae, the dominant intestinal bacteria were Firmicutes and Proteobacteria. In specific, in 3rd and 4th instar larvae, the main intestinal bacteria were *Staphylococcus*, while in 5th instar, it was *JC017*. For the prediapause larvae, the dominant phylum in 3rd instar larvae was Firmicutes, with the dominant genus of *Staphylococcus*, while in 4th and 5th instar larvae was Bacteroidota, with the dominant genus 4th instar was *Staphylococcus*, and in 5th instar was *JC017*. KEGG functional prediction analysis revealed that functional bacterial groups involved in metabolism had the highest abundance values. Specifically, the amino acid metabolism of metabolism-related functional genes in the 3rd instar prediapause larvae was significantly lower than that in the 4th and 5th instar prediapause larvae and the non-diapause treatment. However, the carbohydrate metabolism in 3rd instar prediapause larvae was significantly higher than that in 4th and 5th instar prediapause larvae and non-diapause treatments. The dominant bacterial phylum in the prediapause larvae at different stages of *L. sticticalis* was varied, and there were significant differences in community diversity and richness.

**Discussion:**

These results suggest a complex interaction between the hosts’ physiological state and its gut microbiota, indicating that bacterial communities may assist insects in adapting to diapause preparation by regulating their metabolic levels. This study lays the foundation for further understanding the physiological mechanisms by which intestinal microorganisms regulate overwintering dormancy in the *L. sticticalis*.

## 1 Introduction

Many insects undergo mandatory or facultative diapause stage during their lifecycle as an adaptive strategy to enhance their ability to survive under adverse environmental conditions (Denlinger, 2002). Mandatory diapause occurs at a specific, genetically determined stage and is entirely regulated by endogenous factors, with its initiation unaffected by external environmental conditions. It is a stable genetic trait, usually occurring once per year. In contrast, facultative diapause is modulated by environmental factors (such as reduced photoperiod) and can occur multiple times within a year (Mushegian and Tougeron, 2019). The diversity of diapause strategies in insects has contributed to their evolution, enabling them to survive in fluctuating environments and adjust stable reproductive stages and generations events. At the same time, diapause is synchronized insects population development and reproduction with favorable seasonal conditions, thereby effectively utilizing seasonal resources (Dittmer and Brucker, 2021). Therefore, the significance of diapause for the continuity of insect populations not only to increasing insects survival at adverse environments, but also promotes the uniformity of population development, enhances the probability of mating between males and females, and ensures the reproduction of population (Li et al., 2018; Morey et al., 2012; Posledovich et al., 2015). Research on insect diapause can provide a crucial theoretical basis for the utilization of beneficial insects and for the management of insect pests (Denlinger, 2002). However, both types of diapause incur costs, potentially impacting longevity and reducing reproductive success (Matsuo, 2006; Munyiri et al., 2004; Williams et al., 2003).

There is an inseparable relationship between insect gut microbiota and their hosts. Through long-term natural selection and coevolution, insects provide habitats for gut microbial communities, and the gut microbiota provide nutritional resources for the insects growth, development, and reproduction (Guevara-Rozo et al., 2020; He et al., 2022; Paniagua et al., 2018). Gut microbiota could also participate in the chemical information exchange and metabolism of insect hosts (Zhao et al., 2019). They also improve host defense and detoxification capabilities; affect the lifespan and development cycle of insect hosts, and regulate hosts mating behavior and reproductive capabilities. Previous studies have demonstrated that the gut microbiota regulates nutrient allocation in *Drosophila melanogaster* by regulating the expression of the insulin/insulin-like signaling pathway (IIS), which is one of the key regulatory factor for insect growth and nutritional homeostasis (Shin et al., 2011). Gut microbiota might also play a role in regulating insect diapause, especially in host nutrient allocation and metabolism (Dittmer and Brucker, 2021; Perrot-Minnot et al., 1996). For example, yeast-like symbiotic bacteria and *Wolbachia* in the brown planthopper *Nilapavata lugens* can provide the host with essential amino acids and vitamin B for growth (Ju et al., 2020). The host organism constitutes the ecosystem of its microbiome (Sicard et al., 2014), and changes in this ecosystem will also affect the microbiome. For instance, some microorganisms may decrease or extinction under diapause conditions, causing other bacterial groups to over proliferate (Perrot-Minnot et al., 1996). Considering the host-microbiome community as a whole system, insect diapause may have huge impacts on the change and maintenance of the interaction between insect host and gut microbiome community (Mushegian and Tougeron, 2019).

The beet webworm *Loxostege sticticalis* is a temperate pest that mainly occurring in the area between 36° to 54° North latitudes. It is a major pest in agriculture and natural grassland in Northern, Northeast and Northwest of China (Huang et al., 2024). The beet webworm *L. sticticalis*, by means of diapause and long-distance migration webworm, all causing huge economic losses and ecological disasters (Huang et al., 2024). The *L. sticticalis* overwinters as mature diapausing larvae in the cocoon. Diapause allows larvae to survival through periods of unfavorable, or extremely harsh, cold, and devoid of potential food conditions, and then safely overwintering for a long period of 8 months (Huang et al., 2009). Diapause in *L. sticticalis* represents a syndrome of developmental, behavioral, eco-physiological, and biochemical attributes that single or together serve to enhance survival during overwinter. Generally, compared with non-diapause larvae, diapause individuals feed more during prediapause, ensure to accumulating more energy materials, such as trehalose, fat, and triglyceride (Hahn and Denlinger, 2011), and their respiratory metabolism decreases significantly during the whole diapause period (Ragland et al., 2011). However, the relationship between energy metabolism, the steady state of cell function during diapause and the regulation of intestinal gut microbiota is still unknown.

In recent years, a wide diversity of gut microbiota was found to colonized in the insects gut, which are now well-known, as its always play key roles in the insect host, by regulating homeostasis and metabolic functions (Engel and Moran, 2013; Resnik-Docampo et al., 2018). Research on the effects of gut microbiota on host physiology, behavior, and coevolution has becoming the frontier research topic (Wilson et al., 2010; Douglas, 2015). The interaction between hosts and its microbiota is extremely comprehensive. However, the study of gut microbiota related to regulating of insect rediapause is still lacking systematic analysis. Therefore, this study employed amplicon sequencing to compare the structure of the bacterial community in the gut of *L. sticticalis* larvae at three instar (3rd, 4th, and 5th), that reared at two different photoperiods, i.e., the diapause preparation condition (a short day-length of LD12:12) and non-diapause induced condition (a long day-length of LD16:8), to analyze the bacterial community and its potential function. This study aims to evaluate the impact of host status, whether in the nondiapause or prediapause stage, on the bacterial community, laying the foundation for further understanding the physiological mechanisms through which gut microbiota regulate overwintering diapause in *L. sticticalis*. It also provides new perspectives and avenues for developing effective control strategies for the *L. sticticalis*.

## 2 Methods

### 2.1 Test insects

The test insects were collected from Hohhot, Inner Mongolia Autonomous Region, China. After being reared for two generations in the laboratory, the third generation were used for the current experiments. The newly hatched larvae were fed with fresh leaves of goosefoot *Chenopodium album*, at a temperature of 21 ± 1°C, a relative humidity of 70%–80%, combined with a photoperiod of L:D = 16:8 to induce nondiapause, a photoperiod of L:D = 12:12 to induce diapause in artificial climate chambers. Each box contained at least 40 individuals and five replicates were used for each treatment, to obtain different developmental instar individuals of prediapause and non-diapause larvae, respectively. The larvae were identified as 3rd, 4th, and 5th instar according to the head capsule width and body length (Hu et al., 2020).

### 2.2 Sample collection

Firstly, the collected larvae were transferred into an ice box for 15 min chilling the larvae were immersed in the 75% ethanol solution for 2–3 min and washed with sterile water for 15–30 s (if the washing solution was turbid, it could be washed 2–3 times), to eliminate the possibility of epidermis microbial residues on the diversity of intestinal bacterial community. For the dissecting of the gut, the excess water of the larvae was absorbed by filter paper, and dissected the larva under a microscope (LEICA M250C), and the midgut was removed from the whole body with sterilized tweezers, and transferred in a 1.5 mL sterile centrifuge tube. Immediately frozen in liquid nitrogen, and store at −80°C for subsequent experiments.

### 2.3 DNA extraction and sequencing

For gut microbial community sequencing, 30 larvae from 3rd instar, 20 larvae from 4th instar, and 10 larvae from 5th instar were dissected. Total genome DNA from samples was extracted using CTAB method. DNA concentration and purity was monitored on 1% agarose gels. The V3–V4 region of the bacterial 16S rRNA gene was PCR amplified using the specific primers 341F (5′-CCTAYGGGRBGCASCAG-3′) and 806R (5′-GGACTACNNGGGTATCTAAT-3′) with barcodes. The PCR reaction system was 30 μL, included 15 μL 2 × Phusion Master Mix, 1 μL each 1 μM/μLPrimer, 10 μL (10 ng) gDNA (1 ng/μL), and 3 μL ddH_2_O. The PCR program consisted of an initial denaturation at 98°C for 1 min, 30 cycles of 98°C for 10 s, 50°C for 30 s, 72°C for 30s, final extension at 72°C for 5 min. Then, the PCR products were purified by 2% agarose gel electrophoresis, and the target bands were recovered and purified using the Universal DNA Purification Recovery Kit (TianGen, China) based on PCR product concentration.

Sequencing libraries were generated using NEB Next^®^ Ultra DNA Library Prep Kit (Illumina, United States) following manufacturer’s recommendations and index codes were added. The library quality was assessed on the Agilent 5400 (Agilent Technologies Co., Ltd., United States). At last, the library was sequenced on an Illumina platform and 250 bp paired-end reads were generated.

### 2.4 Bioinformatics and statistical analysis

Firstly, raw data FASTQ files were imported into the format which could be operated by QIIME2 system using qiime tools import program. Subsequently, demultiplexed sequences from each sample were quality filtered and trimmed, de-noised, merged, and then the chimeric sequences were identified and removed using the QIIME2 DADA2 plugin to obtain the feature table of amplicon sequence variant (ASV) (Callahan et al., 2016). The QIIME2 feature-classifier plugin was then used to align ASV sequences to a pre-trained GREENGENES 13_8 99% database (trimmed to the V3–V4 region bound by the 338F/806R primer pair) to generate the taxonomy table (Bokulich et al., 2018). Any contaminating mitochondrial and chloroplast sequences were filtered using the QIIME2 feature-table plugin.

Rarefaction curves were calculated using the core-diversity plugin within QIIME2. Alpha-diversity of microbial communities was assessed using the Chao1, Shannon index, and Simpson evenness. All statistical analyses were done using SPSS Statistics 27 (IBM, Armonk, NY, United States). The data for each index were subjected to a one-way ANOVA to determine any significant differences, then the LSD method for multiple comparisons. Beta-diversity was visualized using a principal coordinate analysis (PCoA) based on Bray–Curtis matrix, and dissimilarity tests based on adonis (PERMANOVA) were used to evaluate differences among groups (Vazquez-Baeza et al., 2013). Based on the species annotation results, the composition of species in the samples was analyzed using community bar plots. The linear discriminant analysis (LDA) Effect Size (LEfSe) method was used to assess differences in microbial communities using a LDA score threshold of 4 (Love et al., 2014; Mandal et al., 2015; Segata et al., 2011). The potential KEGG Ortholog (KO) functional profiles of microbial communities was predicted with PICRUSt (Langille et al., 2013).

## 3 Results

### 3.1 Species annotation and assessment of gut bacteria in *Loxostege sticticalis* larvae

**Figure 1:**
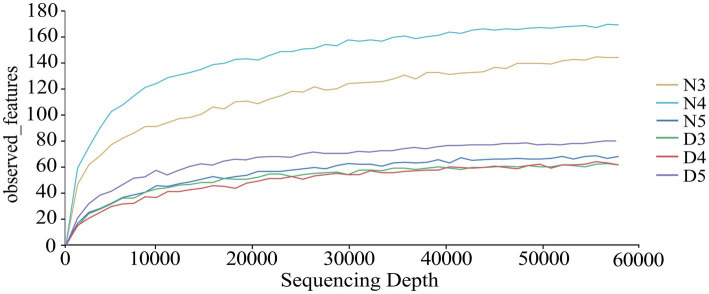
Dilution curves were generated to assess the bacterial composition in the intestines of diapause-preparation and non-diapause larvae of *L. sticticalis*. The following groups were analyzed: D3 (intestinal tract of 3rd instar larvae in diapause-preparation), D4 (intestinal tract of 4th instar larvae in diapause-preparation), D5 (intestinal tract of 5th instar larvae in diapause-preparation), N3 (intestinal tract of 3rd instar larvae in non-diapause), N4 (intestinal tract of 4th instar larvae in non-diapause) and N5 (intestinal tract of 5th instar larvae in non-diapause).

From the 18 midgut samples of non-diapausing and diapause-preparing *L. sticticali*s larvae, a total of 3,178,890 raw sequence reads of the 16S rDNAgene were obtained. After quality filtering and removal of chimeras 2,501,847 high-quality sequences were retained. After rarefying to the minimum sample sequence number, the ASV count was 1,806. Taxonomic annotation of the ASVs revealed a total of 42 phylums, 78 classes, 191 orders, 286 families, 495 generas, and 424 species (Supplementary Table S1). The rarefaction curves of each group leveled off as the sequencing depth increased, indicating that the sequencing depth was sufficient to reflect the microbial diversity (Figure 1).

### 3.2 Diversity of gut bacterial communities in *Loxostege sticticalis* larvae

**Figure 2:**
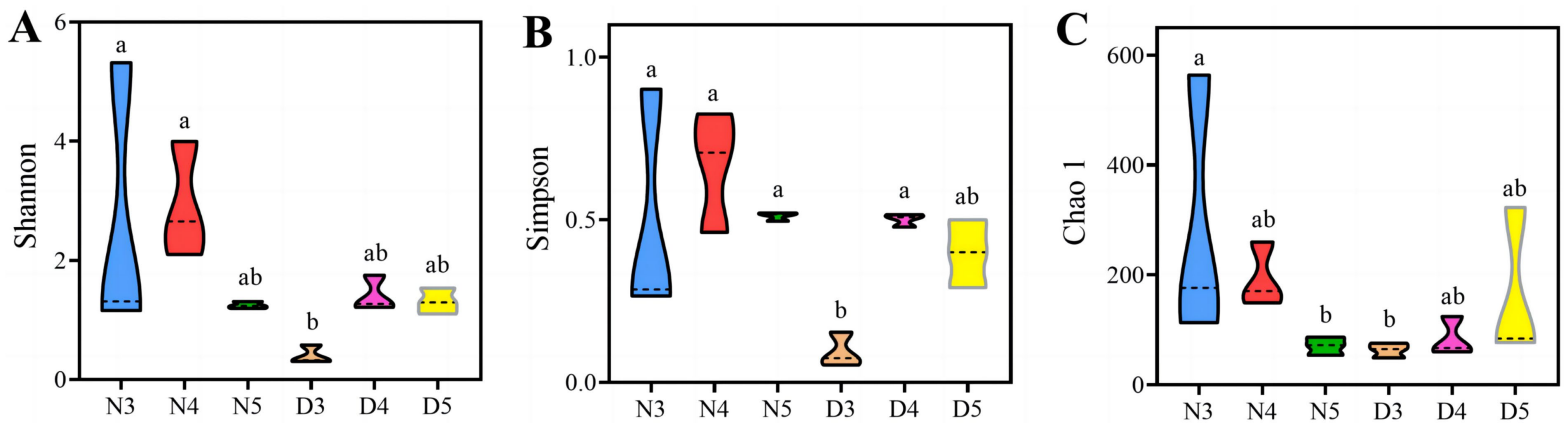
Alpha-diversity of gut bacteria in larvae during pre-diapause and non-diapause stages was assessed using the Shannon index **(A)**, Simpson evenness **(B)** and Chao1 index **(C)**. Different letters above the means indicate significant differences between cultivars (one-way ANOVA, *p* < 0.05).

Alpha diversity analysis results suggest that there are outstanding differences in the gut bacterial alpha diversity indices of *L. sticticalis* under different treatments (*p* < 0.05) (Figure 2). The gut bacterial community diversity index and evenness of 3rd instar larvae during the diapause preparation period were significantly lower (*p* < 0.05) (Figures 2A,B). The gut bacterial community richness of non-diapausing 3rd instar larvae was significantly higher than that of non-diapausing 5th instar larvae and 3rd instar larvae during the diapause preparation period (*p* < 0.05) (Figure 2C).

**Figure 3:**
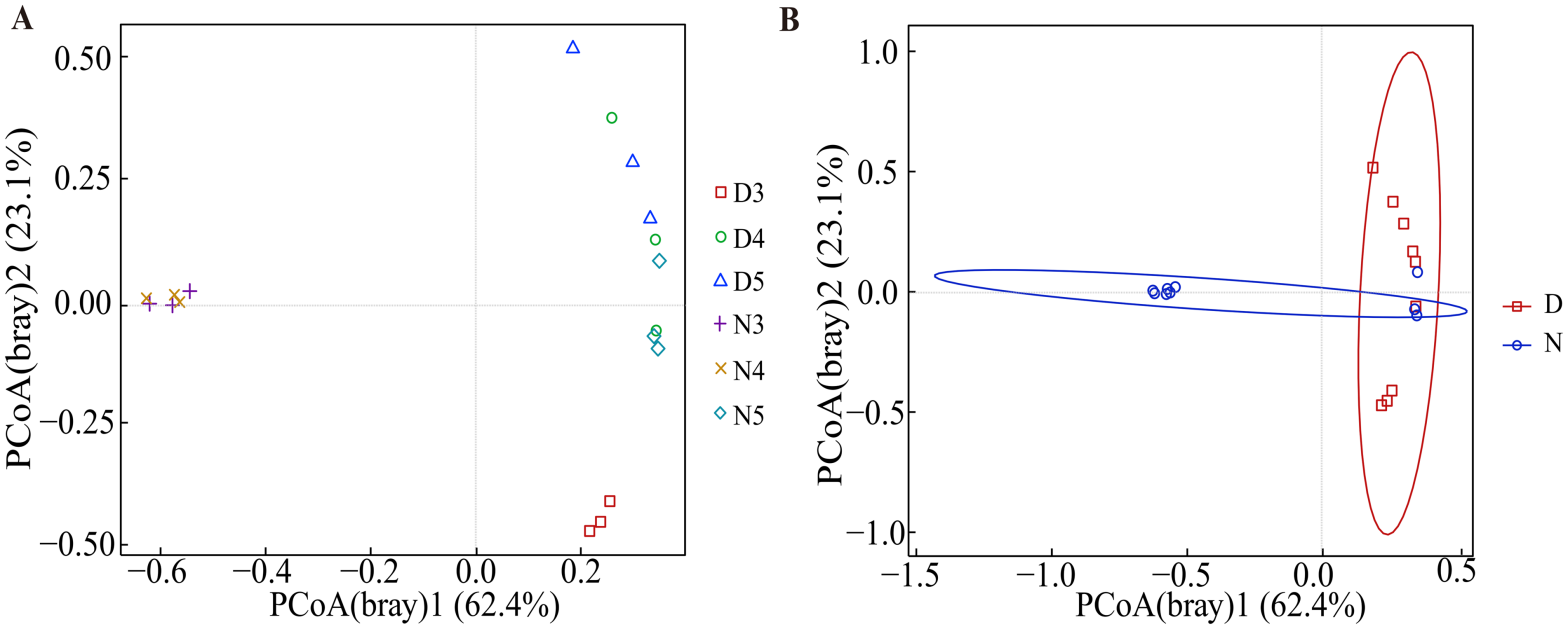
Principal coordinate analysis based on Bray–Curtis distance matrices was conducted on bacterial communities from diapause-preparation and non-diapause *L. sticticalis*. **(A)** Principal coordinate analysis was performed on 3rd, 4th, and 5th instar larvae in both non-diapause and diapause-preparatory stages. **(B)** Principal coordinate analysis was performed on the overall non-diapause and diapause preparation periods. “D” represents diapause-preparation, and “N” represents non-diapause.

The beta diversity analysis results demonstrated that there are differences in the composition of gut microbiota between diapause-prepared larvae and non-diapause larvae (Figure 3 and Supplementary Table S2). Further permutation multivariate analysis of variance was conducted to assess the significance of inter-group differences. The results suggested that there is an outstanding difference in the composition of gut microbiota between diapause-prepared larvae and non-diapause larvae (*p* < 0.05). However, there is no significant difference in the composition of gut microbiota between diapause-prepared and non-diapause larvae at the same developmental stage (*p* > 0.05).

### 3.3 Composition of intestinal bacterial communities in larvae of *Loxostege sticticalis*

**Figure 4:**
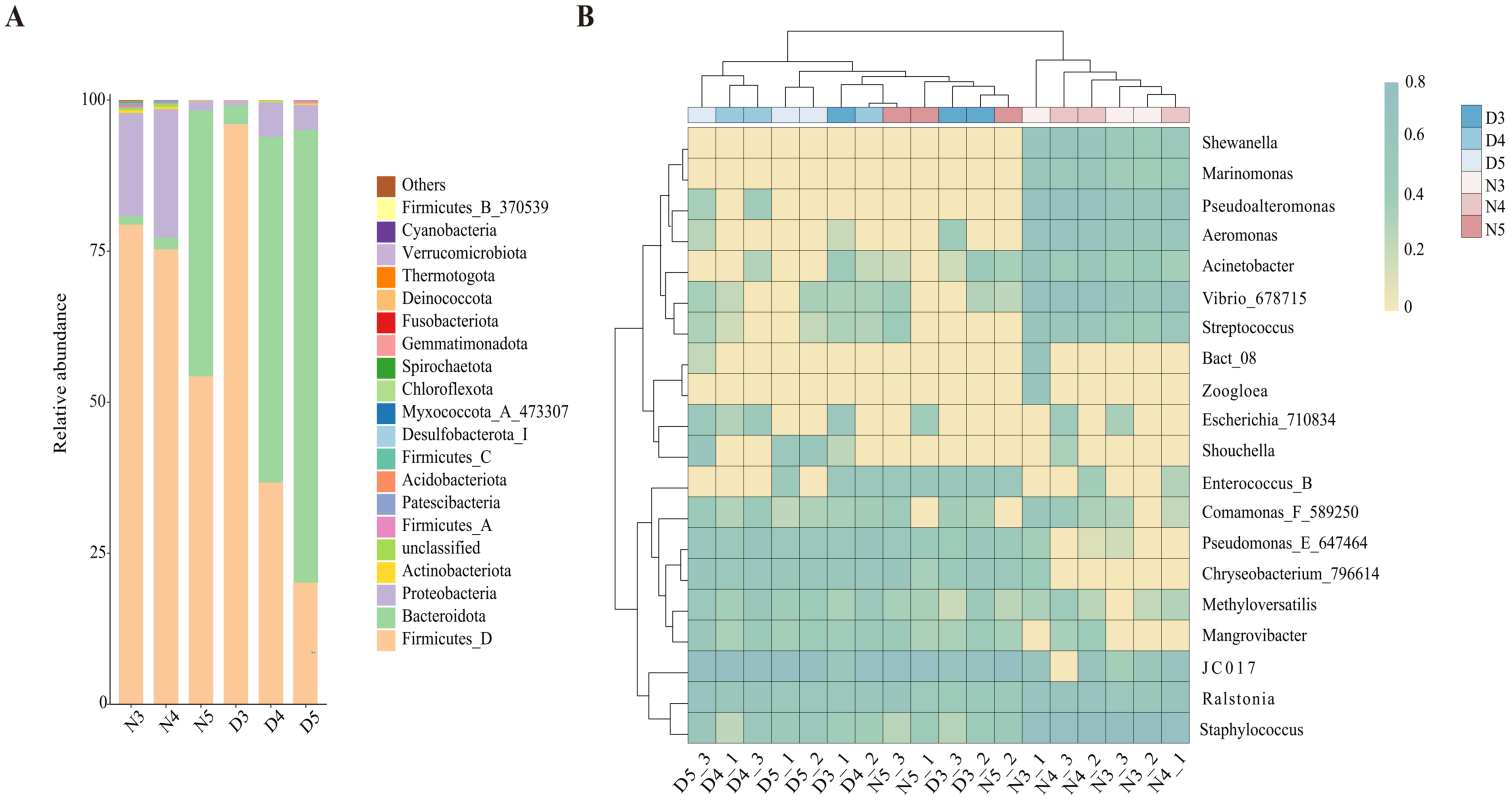
Composition of gut bacteria in diapause-preparing and non-diapause larvae of *L. sticticalis*. The relative abundance of bacterial communities is presented at the phylum **(A)** and genus **(B)** levels. Columns represent different samples, different colors represent distinct bacterial taxa, and “others” represents all species except those annotated above.

At the phylum level analysis of intestinal bacterial communities in larval stages of *L. sticticalis* (Figure 4A), the dominant phylum in the midgut of non-diapausing 3rd and 4th instar larvae is Firmicutes, accounting for 79.4 and 75.3%, respectively, followed by Proteobacteria, accounting for 17 and 21.3%, respectively. In the midgut of non-diapausing 5th instar larvae, the dominant phylum is Firmicutes, accounting for 54.3%, followed by Bacteroidota, accounting for 44%. During the diapause preparation period, the dominant phylum in the midgut of 3rd instar larvae is Firmicutes, accounting or 96%, followed by Bacteroidota, accounting for 3.2%. In the midgut of 4th and 5th instar larvae, the dominant phylum is Bacteroidota, accounting for 57.2 and 75%, respectively, while the subdominant phylum is Firmicutes, accounting for 36.7 and 20.1%, respectively.

At the genus level (Figure 4B), in non-diapausing larvae, the predominant genera in the midgut of 3rd and 4th instar larvae are *Staphylococcus*, while in 5th instar larvae, the predominant genus is *JC017*. Under diapause conditions, the predominant genus in the midgut of 3rd, 4th, and 5th instar larvae is *JC017*. In the midgut bacterial community of non-diapausing 3rd instar larvae, *Enterococcus_B* and *Mangrovibacter* were not detected. Additionally, certain bacterial genera are present in specific larval stages’ midguts, such as *Zoogloea* found in the midgut of non-diapausing 3rd instar larvae, while *Marinomonas* and *Shewanella* are specific to the midguts of non-diapausing 3rd and 4th instar larvae.

**Figure 5:**
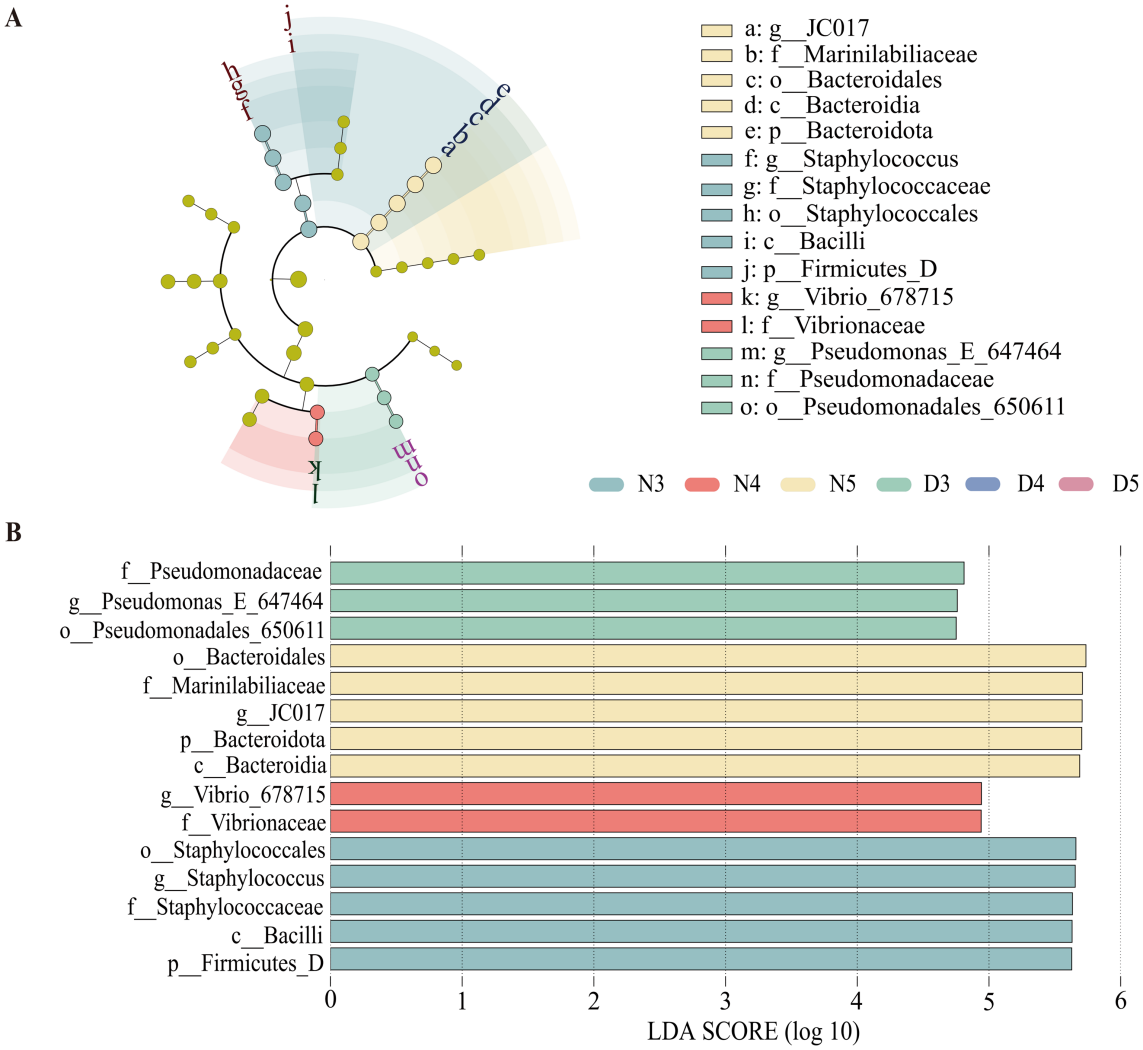
Linear discriminant analysis effect size (LEfSe) analysis of gut bacterial communities in pre-diapause and non-diapause larvae. **(A)** represents the LEfSe analysis bar chart; **(B)** represents the LEfSe analysis cladogram. LDA scored >4.

Cladograms were generated for each gut bacterial and fungal sample, depicting hierarchical relationships from phylum to genus. The analysis identified 15 biomarkers with significant differences between treatments (LDA >4). Notably, both the non-diapause group and the pre-diapause 3rd instar larvae harbored distinct dominant bacterial taxa (Figure 5). At the phylum level, Firmicutes were significantly present in the non-diapausing 3rd instar larvae stage, while Bacteroidota were significantly present in the non-diapausing 5th instar larvae stage. At the class level, Bacteroidia were significantly present in the non-diapausing 5th instar larvae stage, and Bacilli were illustriously present in the non-diapausing 3rd instar larvae stage. At the order level, Bacteroidales were significantly present in the non-diapausing 5th instar larvae stage, Staphylococcales were significantly present in the non-diapausing 3rd instar larvae stage, and Pseudomonadales_650611 were significantly present in the diapause preparation period 3rd instar larvae stage. At the family level, Staphylococcaceae were significantly present in the midgut microbial community of non-diapausing 3rd instar larvae, Vibrionaceae were significantly present in the non-diapausing 4th instar larvae stage, *Marinilabiliaceae* were significantly present in the non-diapausing 5th instar larvae stage, and Pseudomonadaceae were significantly present in the diapause preparation period 3rd instar larvae stage. At the genus level, *Staphylococcus* were significantly present in the non-diapausing 3rd instar larvae stage, *Vibrio_678715* were significantly present in the non-diapausing 4th instar larvae stage, *JC017* were significantly present in the non-diapausing 5th instar larvae stage, and *Pseudomonas_E_647464* were significantly present in the diapause preparation period 3rd instar larvae stage.

### 3.4 Functional prediction of intestinal bacteria 16S in larvae of *Loxostege sticticalis*

**Figure 6:**
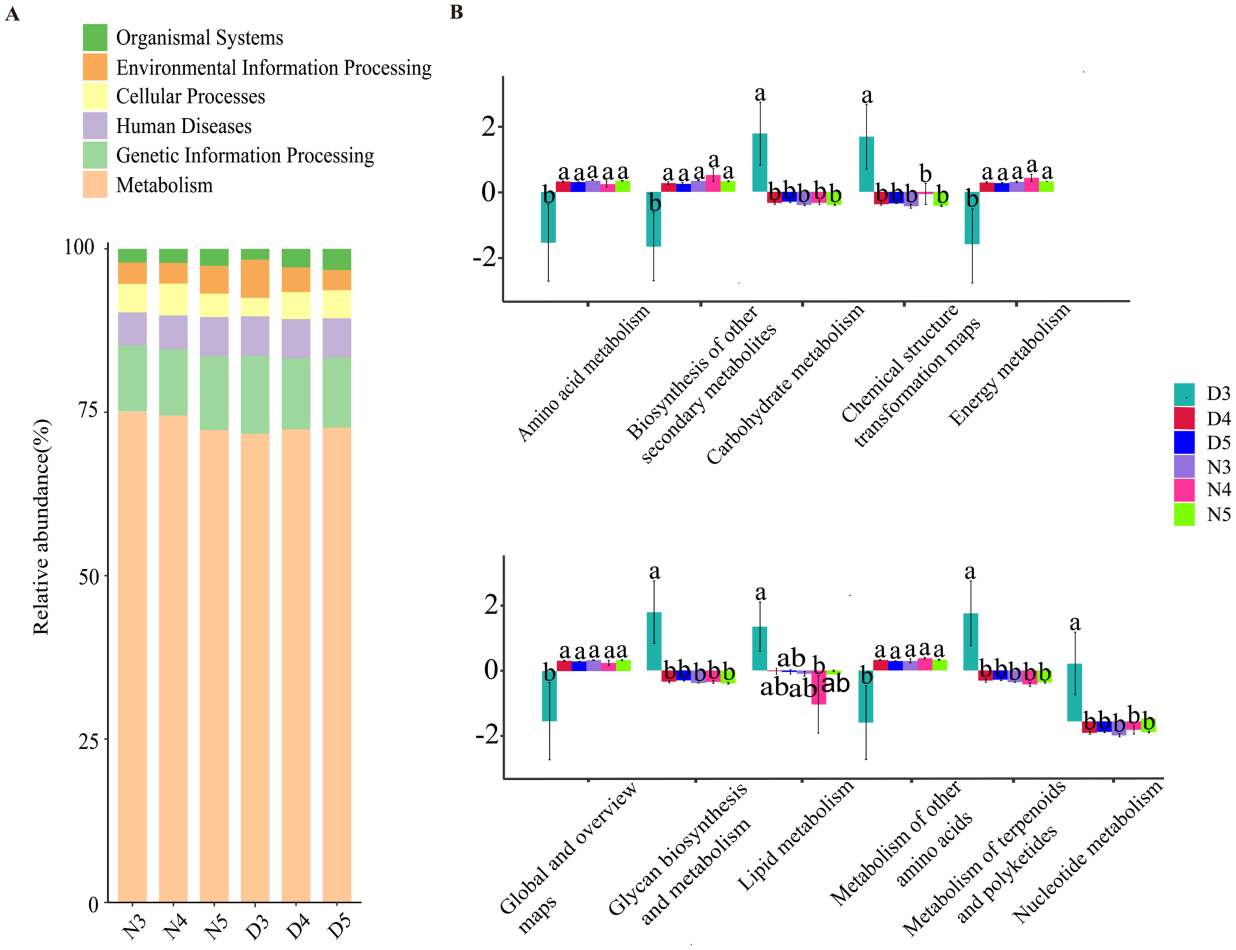
Prediction of intestinal bacterial function in diapause-preparing and non-diapause larvae of the *L. sticticalis*. **(A)** Presents a horizontal stacked chart analysis of first-level classification pathways. **(B)** Illustrates the secondary classification level analysis of metabolic pathways. Different letters above the means indicate significant differences between cultivars (one-way ANOVA, *p* < 0.05).

Based on the information compared to the KEGG database, the Pathway information was obtained, and the abundance of each functional category was calculated based on the ASV abundance. According to the level stacking diagram analysis of the primary classification of pathway, the main pathways include metabolism, genetic information processing, cellular processes, environmental information processing and organic system, among which the metabolic pathway has the highest phase abundance (Figure 6A). The secondary classification level analysis of metabolic pathways demonstrate that the metabolic pathway with the highest abundance of functional flora was amino acid metabolism, followed by biosynthesis of other secondary metabolites, carbohydrate metabolism, chemical structure transformation diagram, and energy metabolism (Figure 6B). The relative abundance of lipid metabolism ranked eighth, indicating a low probability of gut microbes participating in host lipid metabolism. At the same time, found amino acid metabolism, biosynthesis of other secondary metabolites, the single factor variance test carbohydrate metabolism, chemical structure transformation pathways, metabolism, global and overview diagram, glycan biosynthesis and metabolism, lipid metabolism, cofactor and vitamin metabolism, other amino acid metabolism, terpenoid and polyketone metabolism, nucleotide metabolism, and exogenous compound biology degradation and metabolism were different in the six groups. The metabolic functions of intestinal bacteria in amino acid metabolism, biosynthesis of other secondary metabolites, energy metabolism, global and overview maps, terpenes and polyketones of 3rd instar larvae in diapause preparation stage were significantly lower than those of non-diapause larvae and 4th and 5th instar larvae in diapause preparation stage. The carbohydrate metabolism, chemical structure transformation map, nucleotide metabolism, exogenous compound biodegradation and metabolic functions of intestinal bacteria of the 3rd instar prediapause larvae were significantly higher than those of the non-diapause larvae and the 4th and 5th instar prediapause larvae. The lipid metabolism function of intestinal bacteria of the 3rd instar larvae was significantly higher than that of the 4th instar larvae (Figure 6B).

## 4 Discussion

We examined the gut bacterial communities of 3rd, 4th, and 5th instar of nondiapause larvae induced at LD16:8 and prediapause larvae induced at LD12:12 daylength. We found that: (1) gut bacterial communities shown significant differences between nondiapause and prediapause larvae both in alpha diversity and beta diversity; (2) functional gut bacterial groups involved in metabolism had the highest abundance values; (3) gut bacterial communities may assist *L. sticticalis* larvae in adapting to diapause preparation by regulating their metabolic levels. Diapause is the primary strategy by which the *L. sticticalis* survives low temperatures and overwinters safely. Gut microbiota plays a crucial role in regulating the physiological processes in preceding diapause. For one aspect, monitoring shifts in the gut microbial community can help predict transactions in diapause cycles, which may providing an early warning system for pest management. For another aspect, alterations in gut microbiota can impact the immune system of *L. sticticalis*, which may rendering it more susceptible to external pathogens. In the future, microbial modulation technique during insects diapause may become a new direction for more sustainable and eco-friendly pest management.

Insect diapause is a complex developmental process that includes the pre-diapause period (diapause induction and diapause preparation), the diapause period (diapause entry, diapause maintenance, and diapause release), and the post-diapause period (Colinet et al., 2015). Each stage is characterized by significant changes in endocrine activity (Bajgar et al., 2013; Didion et al., 2021; Kucerova et al., 2016), cell proliferation (Shimizu et al., 2018), stress resistance, and nutrient metabolism (Hao et al., 2016; Poelchau et al., 2013; Urbanski et al., 2010). Previous studies have found that glucose and protein levels in *Nasonia vitripennis* larvae increase during diapause, indicating that the microbiome may be involved in diapause-related metabolic remodeling and significantly impact host diapause survival and post-diapause adaptability (Dittmer and Brucker, 2021). Therefore, outsourcing some functions to microorganisms during dormancy may be a strategic adaptation for the host. For example, during reproductive diapause in shield bugs, uricase activity appears to be mediated by Enterobacter-like gut bacteria. Eliminating these bacteria leads to a reduction in amino acids in the hemolymph and results in the rapid death of the host (Kashima et al., 2006). Nitrogenous waste recycling was similarly carried out by bacteria in the guts of hibernating frogs (Wiebler et al., 2018). In hibernating mammals, the composition of the microbial community during diapause shifts to become dominated by bacterial taxa that feed on host epithelial mucins (Dill-McFarland et al., 2014). In this study, we analyzed changes in the gut microbiota of non-diapause and pre-diapause 3rd, 4th, and 5th instar larvae. The results revealed that in pre-diapause 3rd instar larvae, the relative abundance of Firmicutes increased, while Bacteroidetes became a sub-dominant phylum, and the dominant gut bacterial genus shifted from *Staphylococcus* to *JC017*. Additionally, the genus *Enterococcus_B* appeared in the midgut microbiota during this stage. Functional prediction analysis showed that pre-diapause 3rd instar larvae have significantly higher carbohydrate metabolism compared to other instar stages, while amino acid metabolism is significantly lower.

Previous research has demonstrated that the gut microbiota plays a crucial role in the nutritional metabolism of insect hosts (Jing et al., 2020). The phylum Bacteroidetes plays a crucial role in the biosynthesis of essential amino acids and vitamins and maintains host gut health through processes such as polysaccharide metabolism and carbohydrate fermentation (Chang et al., 2000; Jing et al., 2020; Wang et al., 2020). Therefore, we hypothesize that the increased abundance of Bacteroidetes in pre-diapause 3rd instar larvae may be related to the increased demand for essential amino acids and vitamins during the pre-diapause period, aiding the larvae in maintaining normal physiological functions. On the other hand, Firmicutes may enhance the larvae’s ability to digest and metabolize these nutrients through its role in carbohydrate metabolism. This improvement can help the larvae synthesize and store energy, preparing them for the forthcoming diapause stage.

The presence of the genus *Enterococcus_B* may be closely related to immune regulation. Studies have suggested that the main bacterial community in the midgut of *Galleria mellonella* larvae is *Enterococcus* (Kong et al., 2023), and the increase in the number of *Enterococcus* is positively correlated with the increase in the expression level of immune-related genes (Krams et al., 2017). Related studies have also found that *Enterococcus* bacteria play an important role in protecting insects from external damage. *Enterococcus* bacteria can produce bacteriocins, active antibacterial compounds, and can also play a role in the hydrolysis of cellulose (Dantur et al., 2015; Kwaadsteniet et al., 2006). Therefore, in pre-diapause 3rd instar larvae, the increase in *Enterococcus* may be related to an immune response to environmental stress, aiding the larvae in coping with adverse conditions.

Additionally, during diapause in the *Clanis bilineata tsingtauica*, the amino acid metabolism pathways of the gut microbiota are upregulated with the prolongation of diapause, whereas the abundance of Enterococcus shows an opposite trend (Qian et al., 2024). A similar phenomenon has been observed in the *Bombyx mori* (Li et al., 2022). Liang et al. (2022) suggested that *Enterococcus* may maintain the nutritional balance of diapause insects by regulating amino acid metabolic pathways. Therefore, we hypothesize that the reduced amino acid metabolism level in 3rd instar larvae during the pre-diapause stage may be a result of regulation by *Enterococcus*, aimed at conserving amino acids for nutritional needs during diapause.

The results of beta diversity analysis suggested (Figure 3 and Supplementary Table S2) that the species composition structure of the intestinal flora of larvae in the diapause preparation stage and those in non-diapause stage were quite different. Alpha diversity analysis (Figure 2) demonstrated that the diversity index and evenness of the intestinal bacterial community of 3rd instar larvae in the diapause preparation period were significantly reduced (*p* < 0.05). This suggests that some bacterial taxa may be lost during the early stages of diapause preparation. During hibernation, bacterial taxa that rely on dietary polysaccharides decrease in abundance in the mammalian gut, whereas bacteria that degrade host-derived nutrients increase in abundance (Carey et al., 2013; Dill-McFarland et al., 2014). Compared with the 3rd instar larvae, the diversity of intestinal bacteria increased in the 4th and 5th instar larvae during the diapause preparation period. Studies have shown that the abundance of a few bacterial genera increases during diapause, such as *Terriglobus*, which thrives in low temperatures and nutrient-poor environments (Eichorst et al., 2007; Rawat et al., 2012) and *Arsenophonus nasoniae* (Darby et al., 2010; Nadal-Jimenez et al., 2019), a symbiont kills males. The increase in these taxa may be related to their metabolic diversity, the availability of new ecological niches due to the reduction of diapause-sensitive taxa, or the reduction of host regulatory mechanisms (Brucker and Bordenstein, 2014).

## 5 Conclusion

This study demonstrated that: (1) there are differences in the intestinal bacterial communities between larvae in the diapause preparation stage and those in the non-diapause stage; (2) the abundance of gut bacterial communities involved in metabolism was highest during the preparation for diapause; and (3) there are complex interactions between the host and the intestinal microbiota. These findings lay a foundation for further understanding the physiological mechanisms by which intestinal microbes regulate the preparation of overwintering dormancy in the *L. sticticalis*.

## Data Availability

The original contributions presented in the study are publicly available. This data can be found here: http://www.ncbi.nlm.nih.gov/bioproject/1167676.
