# Supplementary material for: Comparison of distinct gut bacterial communities in different stage of prediapause and nondiapause larvae in Loxostege sticticalis
Source: Front Microbiol. 2024 Oct 14;15:1469140. doi: 10.3389/fmicb.2024.1469140 (PMC11513625; doi:10.3389/fmicb.2024.1469140)
Supplement: Supplementary file 1 [file Table_1.DOCX]

Supplementary Table 1. Statistical Analysis of Midgut Bacteria at Various Taxonomic Levels in Diapause Preparation and Non-Diapause Larvae of *L. sticticalis*

| Sample | Phylum | Class | Order | Family | Genus | Species | Num of ATVs |
| --- | --- | --- | --- | --- | --- | --- | --- |
| D3_1 | 8 | 11 | 30 | 41 | 44 | 26 | 76 |
| D3_2 | 10 | 11 | 23 | 29 | 32 | 22 | 65 |
| D3_3 | 6 | 7 | 22 | 28 | 26 | 17 | 49 |
| D4_1 | 9 | 10 | 21 | 26 | 28 | 17 | 60 |
| D4_2 | 13 | 17 | 33 | 38 | 37 | 20 | 67 |
| D4_3 | 12 | 15 | 35 | 42 | 54 | 33 | 124 |
| D5_1 | 7 | 9 | 28 | 34 | 40 | 20 | 84 |
| D5_2 | 7 | 8 | 23 | 31 | 33 | 22 | 77 |
| D5_3 | 18 | 23 | 74 | 109 | 170 | 110 | 323 |
| N3_1 | 35 | 63 | 132 | 177 | 219 | 163 | 556 |
| N3_2 | 10 | 13 | 28 | 35 | 31 | 26 | 113 |
| N3_3 | 6 | 8 | 25 | 37 | 45 | 42 | 171 |
| N4_1 | 8 | 11 | 28 | 42 | 47 | 39 | 170 |
| N4_2 | 9 | 12 | 35 | 52 | 57 | 50 | 259 |
| N4_3 | 10 | 11 | 28 | 36 | 39 | 40 | 149 |
| N5_1 | 9 | 10 | 34 | 38 | 39 | 26 | 72 |
| N5_2 | 8 | 10 | 18 | 22 | 20 | 9 | 54 |
| N5_3 | 11 | 14 | 32 | 46 | 55 | 37 | 87 |
| Total | 42 | 78 | 191 | 286 | 495 | 424 | 1806 |

Supplementary Table 2 Analysis of Variance in Principal Coordinates of Gut Bacteria in Diapause Preparation and Non-Diapause Larvae of *L. sticticalis.*

| Compare groups | | Sample size | Permutations | *P*-vale |
| --- | --- | --- | --- | --- |
| D | N | 18 | 999 | 0.009 |
| N3 | N4 | 6 | 999 | 0.607 |
|  | N5 | 6 | 999 | 0.089 |
|  | D3 | 6 | 999 | 0.093 |
|  | D4 | 6 | 999 | 0.094 |
|  | D5 | 6 | 999 | 0.092 |
| N4 | N5 | 6 | 999 | 0.108 |
|  | D3 | 6 | 999 | 0.11 |
|  | D4 | 6 | 999 | 0.107 |
|  | D5 | 6 | 999 | 0.094 |
| N5 | D3 | 6 | 999 | 0.091 |
|  | D4 | 6 | 999 | 0.219 |
|  | D5 | 6 | 999 | 0.099 |
| D3 | D4 | 6 | 999 | 0.092 |
|  | D5 | 6 | 999 | 0.104 |
| D4 | D5 | 6 | 999 | 0.514 |

Note：D: Diapause preparation period, N：Non-diapause, D3: intestinal tract of 3rd instar larvae in diapause-preparation, D4: intestinal tract of 4th instar larvae in diapause-preparation, D5 :intestinal tract of 5th instar larvae in diapause-preparation, N3: intestinal tract of 3rd instar larvae in non-diapause, N4: intestinal tract of 4th instar larvae in non-diapause, N5: intestinal tract of 5th instar larvae in non-diapause.
